# Multisegment Intradural Extramedullary Ependymoma

**DOI:** 10.7759/cureus.20329

**Published:** 2021-12-10

**Authors:** Rishika Trivedi, Pankaj Trivedi

**Affiliations:** 1 Medicine, Himalayan Institute of Medical Sciences, Dehradun, IND; 2 Medicine, Vasal Hospital Neuro and Trauma Centre, Jalandhar, IND; 3 Neurosurgery, Vasal Hospital Neuro and Trauma Centre, Jalandhar, IND

**Keywords:** low back pain, spinal ependymoma, laminectomy, conus medullaris, intradural extramedullary spine tumors

## Abstract

Ependymomas are commonly reported at an intradural intramedullary location and more frequently at the conus medullaris or filum terminale. In comparison to this, the incidence of spinal tumors being reported at an intradural extramedullary site is less. We describe a young patient who presented with urinary retention and a long-standing history of back pain radiating to the right lower limb. Imaging revealed an intradural ependymoma extending from D11 to S1 and measuring 21 cm in length. The patient underwent D10 to S1 laminectomy. Although the tumor originated from the conus medullaris, the histological evaluation revealed a WHO grade II ependymoma, which is rare, as only 30% of tumors in this location are non-myxopapillary.

## Introduction

Ependymomas are tumors that originate from the ependymal cells lining the ventricles and central canal of the spinal cord. These are slow-growing tumors, with the majority being intramedullary in location. Only 30% of the spinal tumors are reported as intradural extramedullary (IDEM) [1-2]. This paper presents a unique case of IDEM ependymoma involving the dorso-lumbosacral spine region and measuring an extraordinary length of 21 cm. Furthermore, this tumor is a non-myxopapillary ependymoma (WHO grade II) arising from the conus medullaris, which makes this a rare presentation. This variant accounts for only 30% of all ependymomas in this area [3-4].

## Case presentation

**Figure 1:**
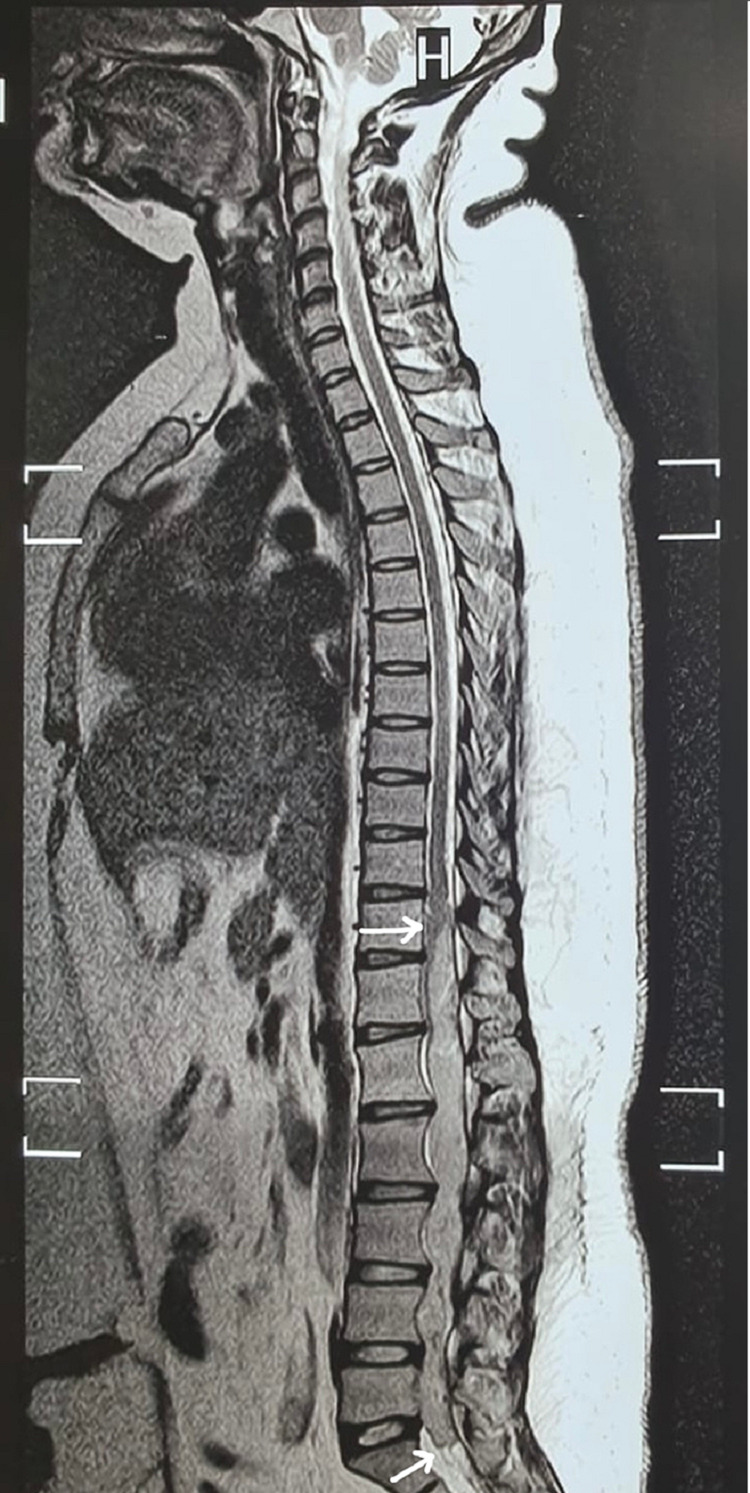
Sagittal T2-weighted image of the spine showing a space-occupying lesion extending from the D11 to S1 segments

A 27-year-old female visited the hospital with a complaint of retention of urine for one day. The patient also had a history of low back pain radiating to the right leg, which developed gradually over eight years. She also noted this pain at rest and developed lower limb weakness during the same period. The pain significantly reduced the walking distance. Her past medical history was unremarkable. Neurologic examination revealed decreased strength in the right leg (MRC grade - 3/5). The tone of the anal sphincter was normal. No significant finding was noted in the blood tests. Magnetic resonance imagining (MRI) of the whole spine revealed a large, space-occupying, intradural spinal lesion extending from the D11 to S1 segments (Figure 1).

**Figure 2:**
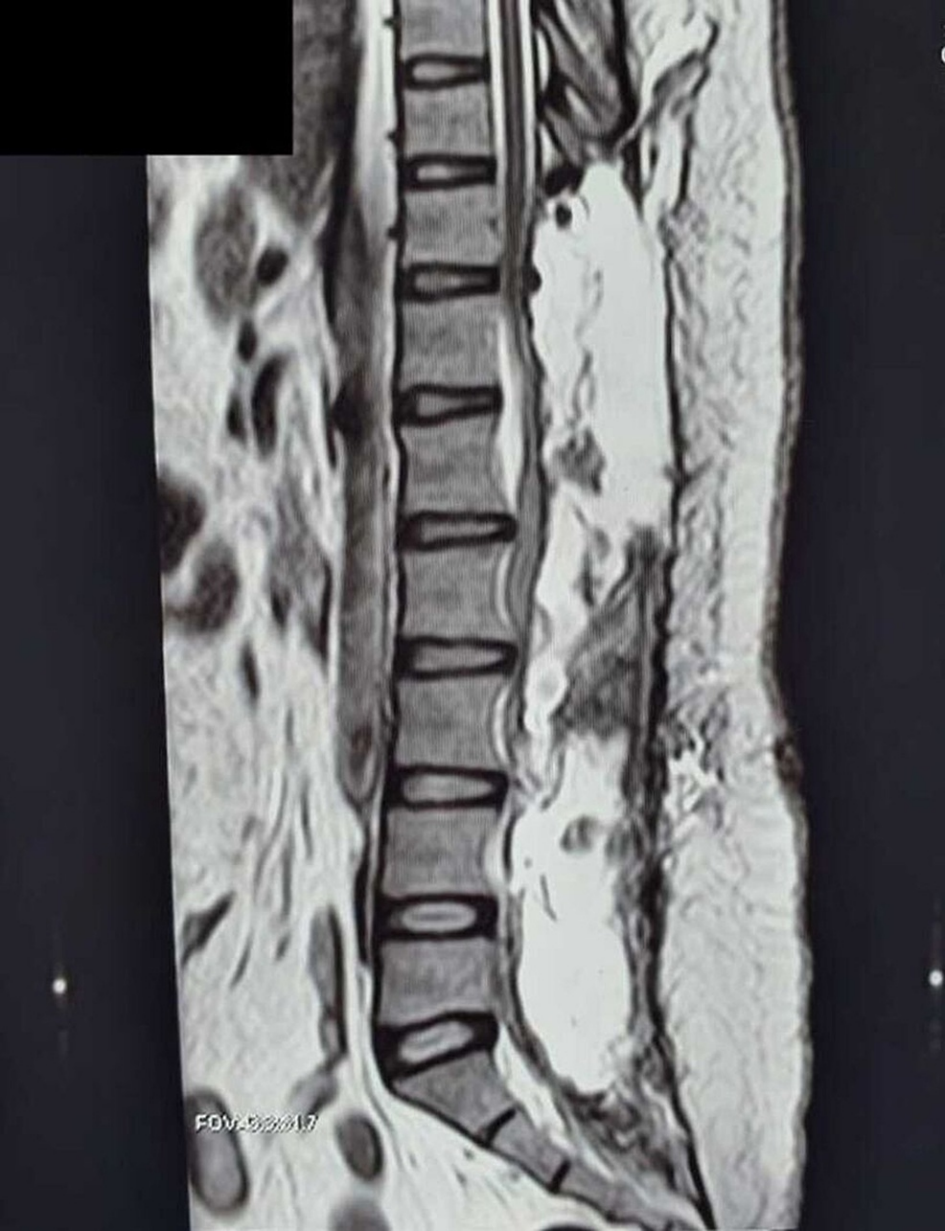
Sagittal T2-weighted image of spine showing removal of the tumor

The patient was catheterized and underwent laminectomy from D10 to S1. This allowed intradural exploration and removal of the mass. The tumor was attached to the conus medullaris. It was carefully removed and was duly confirmed by the postoperative MRI (Figure 2). 

**Figure 3:**
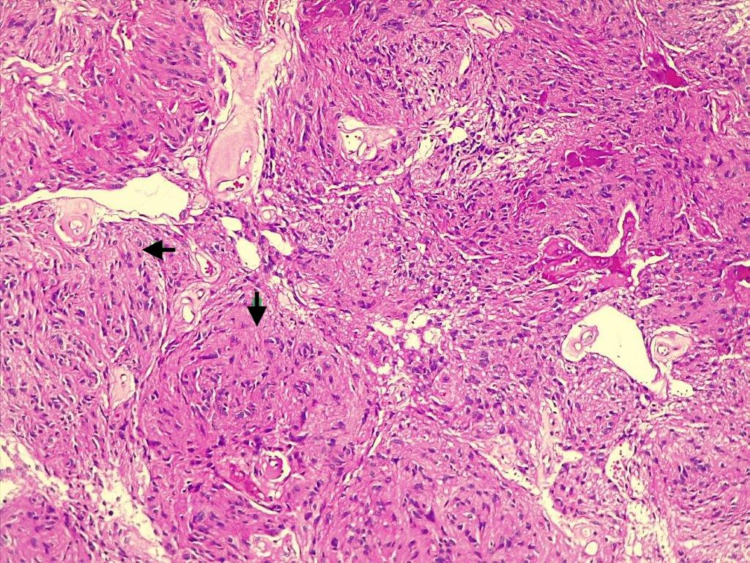
Non-myxopapillary grade II ependymoma: perivascular pseudorosettes with no features unique to the myxopapillary subtype such as mucin

Histopathological examination revealed pseudo-rosettes characteristic of World Health Organization Grade II ependymoma but lacked features unique to the myxopapillary subtype such as mucin (Figure 3) [5].

The patient tolerated the procedure well and was discharged from the hospital in a stable condition.

## Discussion

Primary spinal cord tumors account for 2%-4% of central nervous system neoplasms. They are classified as extradural, intradural extramedullary, and intramedullary based on their anatomic location [6]. However, only 30% of spinal tumors are intradural extramedullary (IDEM) [2].

Ependymomas are neuroepithelial tumors that originate from the ependymal lining of all central nervous system compartments. They are classified as grade I, II ‘classic,’ and III ‘anaplastic’ by WHO on the basis of histopathology [7]. Ependymomas are commonly reported at an intradural intramedullary location and more frequently at conus medullaris or filum terminale. Only a limited number of cases of IDEM ependymomas have been found [8]. Histologically, most ependymomas in the region of conus medullaris and filum terminale are myxopapillary, due to the close proximity to the conus medullaris, these tumors have caused significant postoperative bladder and bowel involvement [9]; merely 30% of tumors in this region are non-myxopapillary [3-4]. Hence, this is a rare case of IDEM ependymoma originating from conus medullaris but presenting as WHO grade II ependymoma, non-myxopapillary on histology.

IDEM ependymoma is a rare tumor with a higher incidence reported in women and commonly encountered in adults [10]. They occur more frequently in the thoracic region and are often described as single-mass tumors [11]. Our literature search found only a few reports of IDEM ependymomas, where the tumor showed extensive thoracolumbar and sacral involvement [12]. Most cases of IDEM ependymomas present with progressive myelopathy as well as pain and weakness according to the tumor location [13]. A similar presentation was noted in this case.

The management focuses on the removal of the tumor via gross total resection (GTR) and preserving the healthy tissue. The removal depends on the location, grade, histology, and presence of a tumor capsule. Since spinal cord ependymomas rarely infiltrate the spinal cord, the rate of GTR in these is as high as 84%-93% [9,14-16]. In a study done by Oh et al., the association between clinical outcomes and histologic grading of spinal cord ependymomas in patients was done, which showed patients with grade II ependymomas who underwent GTR had a significantly lower recurrence rate [14].

In the case presented here, the patient underwent laminectomy from D10 to S1, and careful surgical resection was performed to separate it from the conus medullaris. Postoperative MRI confirmed the removal of the tumor. On a six-months follow-up, there was no sign of recurrence and the patient had no neurological deterioration. The patient will be followed long-term with an enhanced MRI to exclude relapses [17].

## Conclusions

We report a rare case of multisegment IDEM ependymoma measuring 21 cm originating from conus medullaris and extending over the dorso-lumbar and sacral regions. Even though ependymomas situated in this region are commonly reported as myxopapillary, the tumor in the current case revealed a non-myxopapillary variant (WHO grade II) on histological examination. The tumor was resected via D10 To S1 laminectomy without any deterioration of neurological functions. There have been no recurrences on a six-month follow-up.
